# Accelerating Scientific Discovery Through Computation and Visualization II

**DOI:** 10.6028/jres.107.019

**Published:** 2002-06-01

**Authors:** James S. Sims, William L. George, Steven G. Satterfield, Howard K. Hung, John G. Hagedorn, Peter M. Ketcham, Terence J. Griffin, Stanley A. Hagstrom, Julien C. Franiatte, Garnett W. Bryant, W. Jaskólski, Nicos S. Martys, Charles E. Bouldin, Vernon Simmons, Oliver P. Nicolas, James A. Warren, Barbara A. am Ende, John E. Koontz, B. James Filla, Vital G. Pourprix, Stefanie R. Copley, Robert B. Bohn, Adele P. Peskin, Yolanda M. Parker, Judith E. Devaney

**Affiliations:** National Institute of Standards and Technology, Gaithersburg, MD 20899-001; Department of Chemistry, Indiana University, Bloomington, Indiana 47405; National Institute of Standards and Technology, Gaithersburg, MD 20899-001; Instytut Fizyki UMK, Grudziadzka 5, 87-100 Torun, Poland; National Institute of Standards and Technology, Gaithersburg, MD 20899-001; National Institute of Standards and Technology, Boulder, CO 80305; National Institute of Standards and Technology, Gaithersburg, MD 20899-001; National Institute of Standards and Technology, Boulder, CO 80305; National Institute of Standards and Technology, Gaithersburg, MD 20899-001; National Institute of Standards and Technology, Boulder, CO 80305; National Institute of Standards and Technology, Gaithersburg, MD 20899-001

**Keywords:** discovery science, FEFF, FeffMPI, genetic programming, Hylleraas-Configuration Interaction, immersive environments, Lennard-Jones, nanostructures, screen saver science, parallel computing, QDPD, scientific visualization

## Abstract

This is the second in a series of articles describing a wide variety of projects at NIST that synergistically combine physical science and information science. It describes, through examples, how the Scientific Applications and Visualization Group (SAVG) at NIST has utilized high performance parallel computing, visualization, and machine learning to accelerate research. The examples include scientific collaborations in the following areas: (1) High Precision Energies for few electron atomic systems, (2) Flows of suspensions, (3) X-ray absorption, (4) Molecular dynamics of fluids, (5) Nanostructures, (6) Dendritic growth in alloys, (7) Screen saver science, (8) genetic programming.

## 1. Introduction

**Fig. 1:**
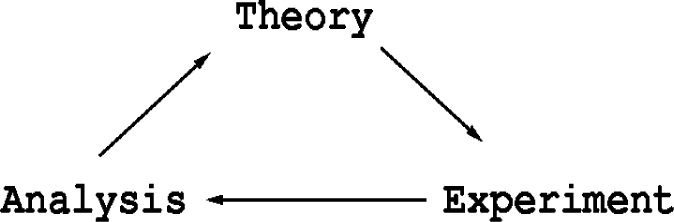
Abstraction of research loop.

The process of research may be abstracted into three major components as shown in Fig. 1. Increasingly *experiment* means computational experiment as computers increase in speed and memory. Parallel computing assists in this by providing access to more processors and more memory. Consequently more complex models that run in feasible times become possible. Laboratory experiments as well are becoming parallel as combinatorial experiments become more common. Both of these lead to large datasets where analysis benefits greatly from visualization.

In this paper we describe research collaborations, between members of the Scientific Applications and Visualization Group (SAVG) and scientists in other labs at NIST, that incorporate parallel computing and visualization as integral parts of the research process. The paper is organized as follows. First, in Sec. 2 we describe our immersive environment. 3D immersion greatly assists in understanding data by literally putting the viewer inside their data. It is used in almost every project we do. In Sec. 3 we describe a computational *measurement* that is not only the most accurate in the world but also extensible to larger systems. Following, Sec. 4 describes computer simulations of complex fluids like suspensions. We present results for concrete. Parallel computations of the near edge structure of clusters of atoms are given in Sec. 5. Replicated data parallelizations of molecular dynamics simulations of fluids are presented in Sec. 6. We describe parallel algorithms for constructing nanostructures as well as visualization of these structures in Sec. 7. Section 8 details our simulation of the solidification process of a binary alloy. Screen saver science, Sec. 9, is our name for our distributed computing project, designed to harvest the cycles of computers across NIST when they are in screen saver mode. Lastly, in Sec. 10, we discuss the NIST genetic programming system. Currently it is being used to automatically generate functional forms for measurement errors.

## 2. Immersive Scientific Visualization

The Immersive Visualization (IV) laboratory plays a key role in accelerating scientific discovery. The NIST scientist views their serial/parallel computation results or experimental data with our IV system. The advanced visualization provided by virtual reality techniques in our IV environment provides greater insight into large, complex data sets by allowing the scientist to interactively explore complex data by literally putting the scientist inside the data.

Fully immersive scientific visualization includes: one or more large rear projection screens to encompass peripheral vision, stereoscopic display for increased depth perception, and head tracking for realistic perspective based on the user’s viewing direction. The NIST IV laboratory is configured as an immersive corner with two 2.44 m × 2.44 m (8 ft × 8 ft) screens flush to the floor and oriented 90 degrees to form a corner. The large corner configuration provides a very wide field of peripheral vision. It is also important for the sense of immersion for the screens to be flush with the floor. The system fulfills the other immersive characteristics with stereoscopic displays and head/wand tracking hardware.

Large and complex data sets are becoming more commonplace at NIST, as high performance parallel computing is used to develop high fidelity simulations, and combinatorial experimental techniques are used in the laboratory. IV is significantly different from traditional desktop visualization and significantly more effective at illuminating such data sets [1]. One analogy for describing this difference is to consider the difference between viewing the fish in a fish bowl and swimming with the fish at their scale. However, the benefits of IV can only be gained when scientists use it. The key ingredient to making IV accessible to scientists is to provide the ability to simply and quickly move their data into the immersive environment.

The primary software controlling the NIST IV environment is an open source system named DIVERSE (Device Independent Virtual Environments—Reconfigurable, Scalable, Extensible) [2, 3]. The DIVERSE API (Application Programming Interface) facilitates the creation of immersive virtual environments and asynchronous distributed simulations by handling the details necessary to implement the immersive environment. The software runs on a variety of display devices from desktop systems to multi-wall stereographics displays with head tracking.

Included with DIVERSE is an extensible application called Diversifly that allows various techniques for navigation through user data loaded from a variety of external data formats. On top of this DIVERSE/Diversifly infrastructure, SAVG has developed additional tools and techniques for quickly moving research data into the IV environment, often with little or no special-purpose graphics programming. The approach used is to apply the classic UNIX^1^ philosophy [4] of creating small and reusable tools that fit into the immersive environment and which lend themselves to combination in a variety of ways to perform useful tasks such as moving the results of a numerical simulation into the immersive environment.

Using this philosophy, SAVG is providing the key ingredient to making IV accessible to NIST scientists by:

- developing simple and reusable graphics file formats implemented as Diversifly file loaders;
- developing application-specific files that are easily transformed into existing or newly created graphics file formats;
- developing filters (scripts, small programs, etc.) to connect data transformation pipelines.

Diversifly is extensible. Distributed Shared Objects (DSOs) can be written for it to implement a new input graphics data format. These DSOs are much like subroutines (C/C++ functions). However, they are linked into the executable code at run time and the main Diversifly application need not be modified or recompiled. For the visualization of ellipsoidal objects (e.g., concrete particles) in suspension discussed in Sec. 4, a file loader DSO was created. This DSO implemented the animation as a time sequence of 3D data. For this new .*seq* file type, each time step of the sequence was made general in order to allow all other possible graphics file types to be used. Thus, not only were the requirements for this application met, but a wide variety of other applications involving animation as a time series of 3D data can be quickly implemented by reusing this DSO.

The development of an application-specific file format doesn’t always require a DSO extension to Diversify. For example, an application that required the display of colored spheres at random locations was satisfied by defining an ASCII file to simply list the *x*, *y*, *z* sphere locations, the sphere diameters, and the corresponding sphere colors. Using a simple scripting language, a filter named *sphere2iv* was created to convert the sphere list format to an Open Inventor [5] file (.*iv*) which is one of the many file formats compatible with DIVERSE. While it is possible for a user to directly write the .*iv* file, the approach followed here allows a user to concentrate on their application by simplifying the graphics data format to essentially what they are already doing. Again, the simplicity of this approach provides quick access to the IV environment for a range of applications requiring the display of colored spheres. Now that this tool has been added to the tool box, it can be combined with the .*seq* file format to produce other types of time series animations.

**Fig. 2:**
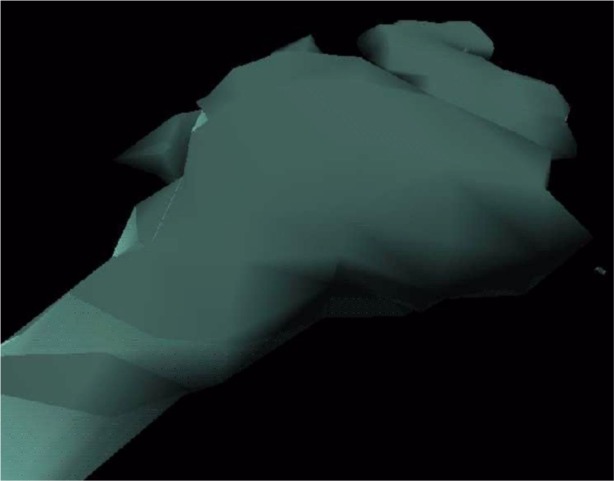
Cloud example.

**Fig. 3:**
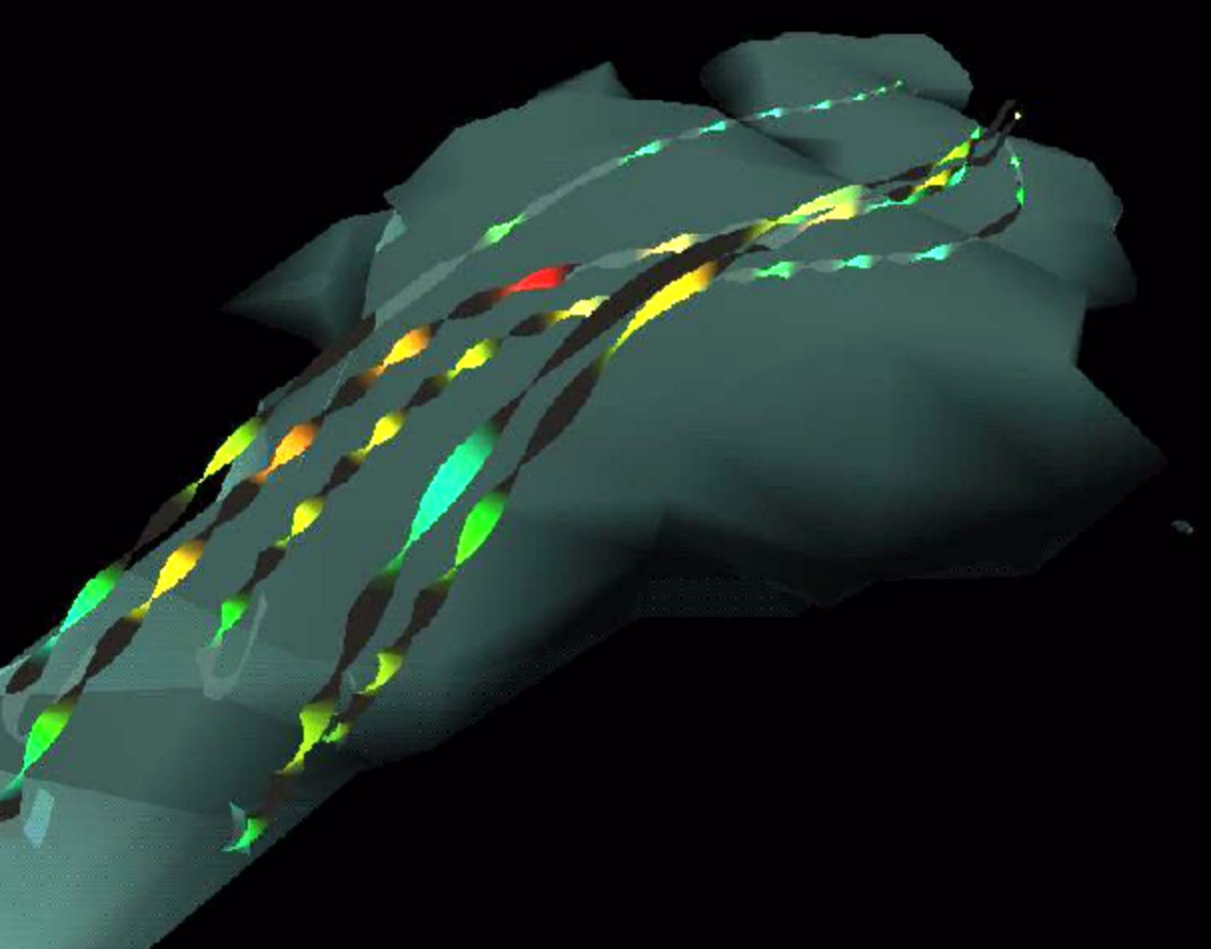
Click to turn on streamlines.

In addition to using DSOs for adding new graphics file loaders, they can also be used to extend the functionality of Diversifly. Using this capability, interactive features can be added. For example, Fig. 2 shows a visualization of a cloud water dataset (cloudwater.dx) derived from a sample dataset and program provided by the Open Visualization Data Explorer (OpenDX) software package [6]. With the addition of a file switcher DSO, an interactive user click can be used to turn *on* (or *off*) streamlines as illustrated in Fig. 3. In this case, the feature is used as a simple *on*/*off* switch. The most appropriate graphics file type can be used for each view of the visualization. Thus very complex immersive visualizations can be created at run time with the addition of this very simple tool.

**Fig. 4:**
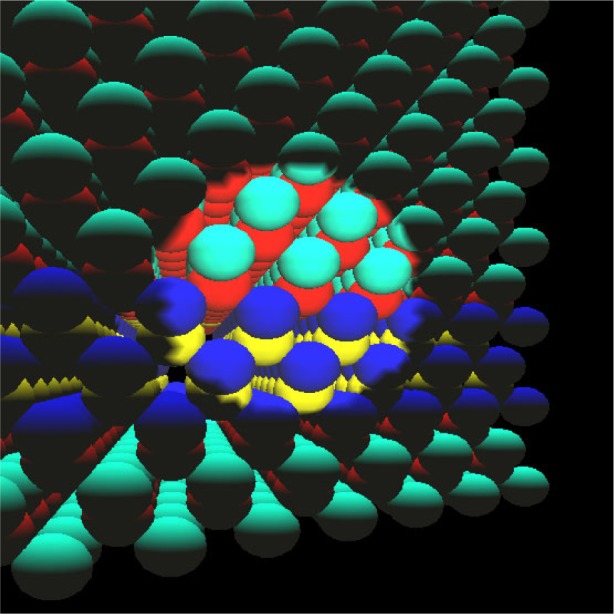
Flashlight DSO enables the user to shine a *flashlight* on a spot *within* the immersive visualization by merely pointing to it.

Another DSO that extends the user interface is a *virtual flashlight*. Figure 4 shows the user view of the virtual flashlight which is attached to the wand. It operates like a hand held flashlight to explore and highlight the virtual world. This capability is extremely useful both for a single user to focus attention on specific areas of the data and for multiple users in a collaborative session. Just like the real world, a flashlight belongs in everyone’s tool box.

**Fig. 5:**
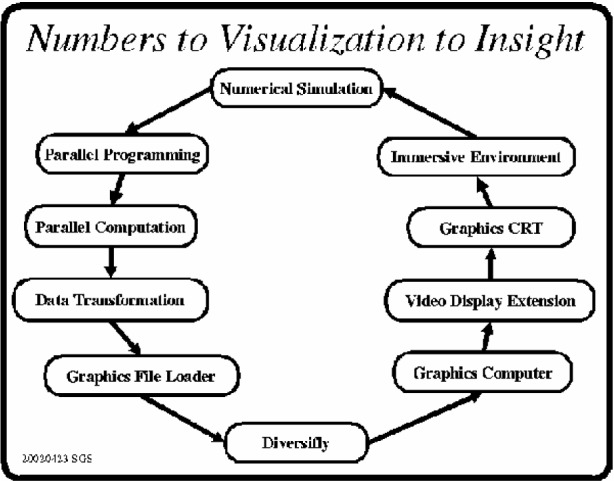
Collaboration process.

The flow diagram in Fig. 5 illustrates how SAVG integrates IV into a research collaboration with NIST scientists which takes the process from numbers to visualization to insight. A typical example will start with a scientist who has developed a model for some scientific research purposes. Often this is implemented as a serial program on a desktop workstation.

Working in collaboration with SAVG staff who specialize in parallel programming, the model is converted to a portable parallel code that can run on a variety of parallel computing platforms. These parallel systems may be shared memory parallel machines, non-shared memory clusters or a combination of both. One obvious advantage of parallelizing the code is that it will typically execute much faster. However, the experience is that scientists have a certain *turn-around* tolerance, meaning, they are willing to wait a certain amount of time for their results. Rather than using the parallel version to run the research problem quicker, the scientists often use the same amount of time to run a bigger, more complex problem that produces more data.

Once numerical results are obtained, the appropriate tools (described above) are pulled out of the tool box to transform the results into data compatible with Diversifly. As necessary, a new tool or application protocol may be developed in order to minimize the impact on the researcher. A very straight forward definition for *User Friendly* is *what the user already knows*. The advantage of the *software tools approach* is that combinations of existing tools with occasional small additions can frequently match the user’s natural data to the visualization data requirements.

After the data is properly transformed, it is then loaded into the Diversifly application which executes on the graphics systems. Since the NIST graphics system is physically located in a secure computer room environment, video display extension hardware is required to transmit the graphics video to the user area where the IV system is located. At this point, the final and most important link in the process is completed when the researcher experiences data in the immersive environment. Here the researcher visually interacts with the data (swims with the fish) and gains some new insight into the research which may then require another loop around the process.

Utilizing a tool box philosophy allows SAVG to visualize large numbers of applications with no programming. For these applications, a toolbox of reusable utilities, data filters, generalized application protocols and data formats that feed into a single, well understood viewing environment can provide almost immediate access to the virtual environment. The potential of immersive visualization to accelerate scientific discovery is realized not only by its ability to provide new insights but also by providing a quick and easy interface to bring science into the immersive environment.

## 3. High Precision Energies for Few-Electron Atomic Systems

Impressive advances have been made throughout the years in the study of atomic structure, at both the experimental and theoretical levels. For atomic hydrogen and other equivalent two-body systems, exact analytical solutions to the nonrelativistic Schrödinger equation are known. It is now possible to calculate essentially exact nonrelativistic energies for helium (He) and other three-body systems as well. Even for properties other than the nonrelativistic energy, the precision of the calculation has been referred to as “essentially exact for all practical purposes” [7], i.e., the precision goes well beyond what can be achieved experimentally. Once the Bethe logarithm for He, long regarded as an unsolved problem, was calculated accurately by Drake and Goldman [8], helium reached the same status as hydrogen in that the lowest order energy and relativistic corrections became essentially exact for all practical purposes. Nevertheless, the scarcity of information on atomic energy levels is overwhelming, especially for highly ionized atoms. The availability of high precision results tails off as the state of ionization and the angular momentum state increases. In addition, atomic anions have more diffuse electronic distributions, and therefore represent more challenging computational targets.

In going from He (two electrons) to Li (lithium, three electrons) to Be (beryllium, four electrons), the situation vis a vis high precision calculations degrades to the point that already at four electrons (Be) there are no calculations of the ground or excited states with an error of less than 10 ^−6^ a.u.^2^ The challenge for computational scientists is to extend the phenomenal accomplishments on He to three, four, and more electron atomic systems.

One might wonder why nonrelativistic energies with uncertainties of 1 × 10^−20^ or better might be desirable. There are essentially two reasons for this.

The first is that the physical energies of interest, e.g., a transition energy or familiar chemical ionization potentials and electron affinities involve these nonrelativistic energies. So the nonrelativistic energies need to be calculated exceedingly accurately to guarantee the accuracy of the result. As one example, consider the first ionization potential for lithium. The first ionization potential, *I*_1_, for the process Li→Li^+^ e^−^, can be determined from [9]

$$I_{1}=E_{\text{NR}}({\text{Li}}^{+})-E_{\text{NR}}(\text{Li})+\Delta E_{\text{REL}}+\Delta E_{\text{MASS}}+E_{\text{QED}}$$ (1)

where the first two terms are the nonrelativistic energies of Li^+^ and Li, respectively, and the remaining three terms refer to the relativistic correction, nuclear mass dependent correction, and quantum electrodynamics (QED) shift, respectively. See King [9] for a more detailed discussion of these terms and for a discussion of transition energies in general. The dominant terms are the nonrelativistic energies. Hence these nonrelativistic energies can be regarded as fundamental atomic data. Once they have been computed “exactly”, they can be used to obtain more and more accurate ionization potentials, electron affinities, etc., as the other (correction) terms become known to increasing precision.

The other reason for seeking a high precision nonrelativistic energy is that when a nonrelativistic energy is obtained, a wave function is also obtained which can be used to compute other physical properties like oscillator strengths, electric polarizabilities, Fermi contact terms, or nuclear mass polarization corrections. Quantum theory tells us that a first order error in the wave function manifests itself as a second order error in the energy [10]. Another way to put this is that the properties are accurate as the square root of the uncertainties of the energy. This can be understood from the fact that the energy is a sum of kinetic and potential energy contributions, so there can be cancellation of errors between the two energy contributions leading to a precision in the energy determination that does not carry over to other properties. Hence the drive for high accuracy calculations.

Modern computing power drastically changes our attitudes towards the variational methods employed. In the case of He, the choices for high accuracy calculations are the following:

1. One that employs a variational expansion in products of exponentials in the problem interparticle distances [11, 12, 13];
2. Standard Hylleraas (Hy) technique [14] calculations as best exemplified by the work of Drake and collaborators [15, 16, 17], which employ factors of powers of the interparticle distance *r_ij_* in the wave function;
3. The combined Hylleraas-Configuration-Interaction (Hy-CI) method [18] in which the wave function is expanded as a linear combination of configurations, each of which contains at most one *r_ij_* to some power.

While the exponential products approach of Korobov [11] leads to a more compact wave function than either of the *r_ij_* techniques for the He ground state and is straightforward to implement, it is not clear how useful it will be for atoms with more than two electrons. From the theoretical work on Li published to date,^3^ it appears essential to incorporate *r_ij_* terms in the wave function, if the highest possible precision is desired. This is not surprising given the monumental two electron calculations of Drake and collaborators [15, 16, 17, 7, 8, 20, 21]. But where Li has been done essentially as accurately as He, it has only been with a 6000 fold increase in CPU requirements to reach spectroscopic accuracy. This may have been what led Clementi and Corongiu [22], in a review article on Computational Chemistry in 1996, to state that using an Hy-CI [18, 23] expansion to solve the dynamical correlation is nearly impossible for more than three or four electrons. While that may have been true in 1996, its validity today is being challenged by the availability of cheap CPUs which can be connected in parallel to enhance by orders of magnitude both the CPU power and the memory that can be brought to bear on the computational task.

For He, the close connection between Hy-*r_ij_* and Hy-CI has been pointed out in a recent article [24]. It seems to us that the Hy-CI method selects the important term types in a more natural manner. Also, the calculation, at least for He, is easier. This, in conjunction with modern computing power, might explain the renewed interest in Hy-CI techniques [25, 26, 27, 28, 29, 20]. It was the impetus for our attempt to come up with a good technique for obtaining very accurate energies for few electron atomic systems using the Hy-CI formalism.

In any attempt to get very accurate energies, large basis sets have to be employed, which means that linear dependence in the basis set is never very far away. To proceed to several thousand terms in a wave function, extended precision arithmetic is needed to obviate the linear dependence problem, which in turn leads to higher CPU costs. The use of several thousand terms in a wave function leads to memory problems arising from storage of the matrix elements prior to the matrix diagonalization step at least for dense matrices that result from the introduction of interelectronic coordinates into the wave function.

The solution to these problems, for both CPU speed and memory needs, is to parallelize the calculation. We now have a working computer program for high precision Hy-CI calculations for the ground state of He and He-like ions [24]. As far as we know, this is the first high accuracy calculation for few electron atomic systems to employ parallel computing. Using the Message Passing Interface (MPI), the problem scales almost perfectly on the 16 node NIST NT cluster where all the parallel runs were done, i.e, 3 hour runs complete in about 10 minutes to 15 minutes.

This working computer program employs a novel wave function, namely, a wave function consisting of at most a single *r*_12_ raised to the first power combined with a conventional configuration interaction (CI) basis. We believe that this technique can be extended to multielectron systems [24], where the use of at most a single *r_ij_* (to the first power) retains the power of *r_ij_* in the wave function without making the integral evaluation overly complicated. We used this technique to determine the nonrelativistic ground state energies for the following members of the He isoelectronic sequence, Li^+^, Be^++^, and B^+3^. Table 1 contains our final energy values in atomic units (a.u.), and compares them with the best previous calculations [31, 20].

The parallel calculations reported here were carried out at the National Institute of Standards and Technology on the instaNT.nist.gov NT Cluster, a 16 node cluster of Pentium II systems running Microsoft Windows NT [Server 4.0] with 100-Mbit Fast Ethernet for interconnection. We used our Microsoft ASM (MASM) extended precision (∼48 digits) package for all of the calculations.

**Fig. 6:**
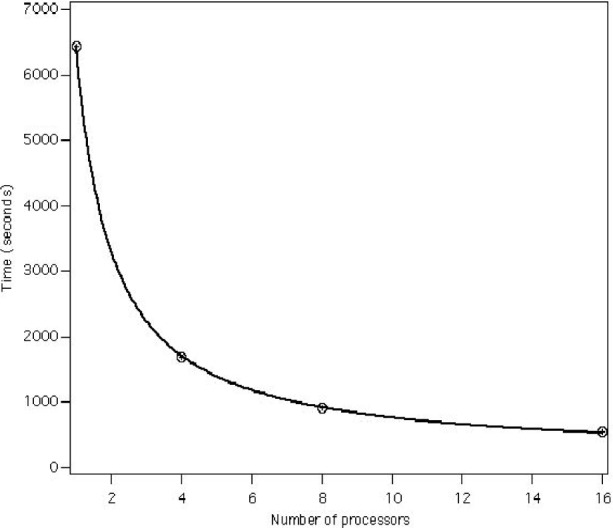
Hy-CI Scaling with Cluster Size.

We found that the processing speed could be predicted, as a function of cluster size, by the simple scaling law *T* = constant (*s* + (1 − *s*)/*N*_proc_), where *T* is the runtime in s, constant = 6419 in this case, and *s* is the inherently sequential part of the calculation. This function is plotted in Fig. 6. We find that the sequential fraction *s* = 0.022, indicating that the scaling is excellent and we could go to a larger number of processors (if we had them).

As we move on to larger systems, computational times will increase greatly, as well as memory requirements, so it is imperative to program these routines using parallel programming techniques and MPI (for portability). If one restricts the wave function to a single *r_ij_* in each term (the *r_ij_* power *n* ≤ 1) then the most difficult integrals are already dealt with at the four electron level and the calculation is greatly simplified. Hence, our goal is to see how well we can do in the four electron case of Be and the isoelectronic Be-like anion, Li^−^.

Li^−^ is interesting because atomic anions have more diffuse electronic distributions (more “open-shell” like character), and therefore represent more challenging computational targets. For example, the He-like isoelectronic anion H^−^ is more difficult than He for this reason. In addition, in the four electron case the “open-shell” like nature of the anion would make the “inter”shell *r_ij_* terms more important than for closed shell atoms like Be.

Li^−^ also is the key to calculating the electron affinity (EA) of the ground state of Li. The EA of Li is the negative of the energy associated with the process Li + e^−^ → Li^−^. Its calculation requires several factors which include the nonrelativistic energy of the neutral and anion ground states, specific mass shifts, Breit-Pauli relativistic corrections, and QED corrections. For the neutral atom all of these quantities are known [32, 9, 33]. For the anion, if we can determine the nonrelativistic energy to high precision, we can make reasonable estimates for the rest. Since Breit-Pauli and QED are smaller than the other terms, reasonable estimates should give satisfactory results. Hy-CI calculations have been done previously for Li^−^ [34]. This result represented for more than 15 years the best upper bound for the ion considered. This result has been considerably improved by Chung and Fulbright [35] and others, culminating in the recent determination by Komasa, Rychlewski, and Jankowski [36]. These authors treat other members of the Be isoelectronic sequence as well, and are the best results for four electron atoms so far. The overall accuracy can be claimed to be on the order of 10^−4^ a.u. for the correlation energies with a nonrelativistic energy uncertainty of 10^−6^ a.u., clearly suggesting the need for higher accuracy calculations. To this end, we have converted our Microsoft ASM (MASM) extended accuracy (∼48 digits) package to IBM AIX assembler language so that we can run on the IBM Teraflop SP System at Indiana University (616 processors). We are also redoing our integral package [23] so that we can bring the power of modern parallel and extended accuracy techniques to bear on this research.

## 4. Computational Modeling of the Flow of Suspensions

The computer simulation of complex fluids like suspensions (e.g., colloids, ceramic slurries and concrete) can be greatly limited by computational costs (such as computer speed and memory), frequently restricting studies to simpler fluids and/or two dimensional systems. To obviate these computational difficulties, we have developed a quaternion-based dissipative particle dynamics (QDPD) approach suitable for a parallel computing environment. The parallelization of the algorithm was done using MPI [37, 38]. The technique, a conventional spatial domain decomposition using a parallel link cell algorithm, has some fairly novel features to accommodate the DPD formalism (which forces some tricky bookkeeping to satisfy Newton’s third law), the use of arbitrarily shaped rigid body inclusions, and the requirement of a sheared boundary condition. A detailed discussion of our implementation will be presented elsewhere [39]. The calculation and subsequent visualization of a sheared suspension in different flow environments is one of the applications treated by this technique.

While various quantitative tests are used to help validate our algorithms, visualization plays an important role in the testing and validation of programs. Even simple visual checks to make sure the rigid bodies satisfy boundary conditions can be helpful.

**Fig. 7:**
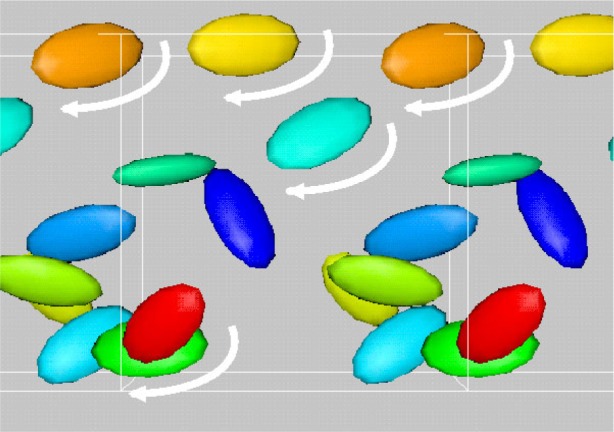
Motion of a suspension of ellipsoids subject to shear. The single ellipsoid rotation is a well known phenomenon called Jeffery’s Orbits.

Figure 7 shows the motion of a suspension of ellipsoids subject to shear. The shearing boundary conditions were obtained by applying a constant strain rate to the right at the top of the figure and to the left at the bottom. Note that all the ellipsoids rotate. This is a well known phenomena seen in experiments called Jeffery’s Orbits. The period of rotation was derived by Jeffery, and our simulations were found to be consistent with this theory, hence serving as a form of validation of our numerical approach.

**Fig. 8:**
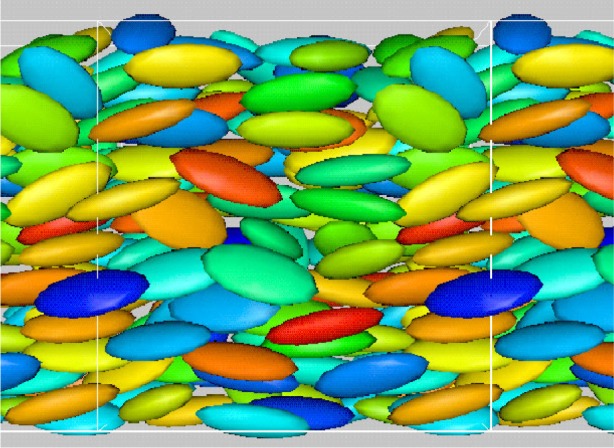
Dense suspension of ellipsoids similar to typical aggregate contribution in concrete. Jeffery’s Orbits are suppressed and the alignment between ellipsoids is enhanced.

In contrast, we found that when many ellipsoidal inclusions were added to the system, and the solid fraction (ratio of solid volume to total volume of the system) of ellipsoids was increased to about 15 % to 20 % (Fig. 8), the Jeffery’s Orbits were suppressed and the ellipsoids had a tendency to align as their relative motion was partly stabilized by mutual hydrodynamic interactions. An interesting consequence of this alignment is that the viscosity of the suspension is lower than that of an equivalent sphere system (same number and volume of spheres). Note that in a dilute concentration regime (Fig. 7), an equivalent suspension of spheres has a lower viscosity. One way to think about it is that once the ellipsoids align it is “easier” for them to get around each other. Hence, the viscosity decreases.

Not only does the visualization of data play an important role in the validation of computer algorithms and the correctness of the physical ideas used, but visualization can lead to ideas about new phenomena that might not be deduced from the enormous data sets created during a simulation. Also, visualization can help the researcher design better numerical tests or come up with other, better ways to evaluate the physical behavior of systems. For example, the strong ordering seen in the simulation of ellipsoids under shear (Figure 8 above) at high solid fractions was unexpected and led to improved ways of quantifying such phenomena.

**Fig. 9:**
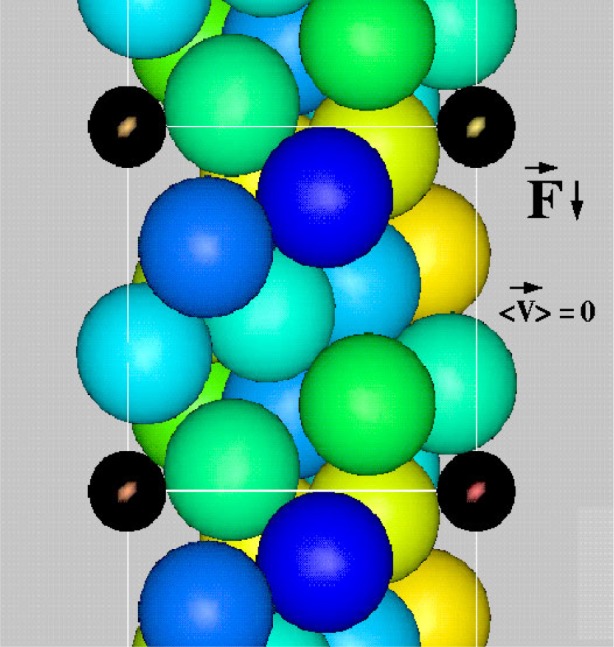
Spherical aggregates subject to a downward applied force. Sphere diameter is approximately one-half the gap spacing between reinforcing rebars. The “jamming” of the aggregates between cylindrical rebars (in black) is observed.

**Fig. 10:**
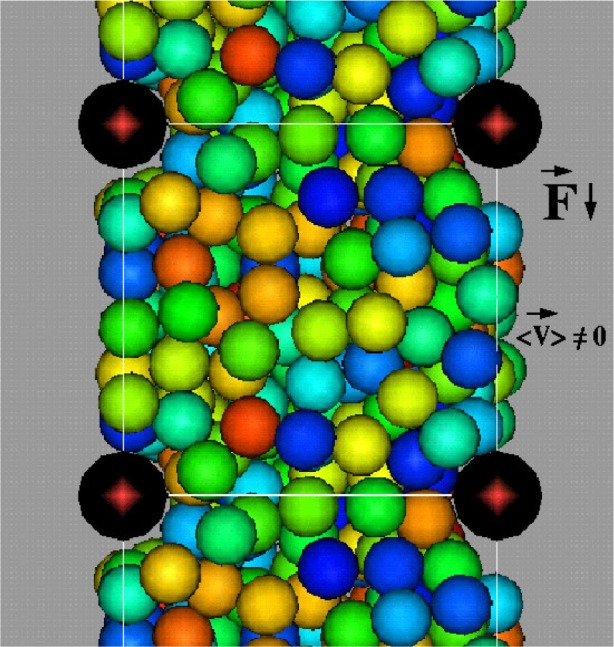
Spherical aggregates moving around stationary cylindrical rebars (in black). Sphere diameter is one-fifth the gap spacing. No “jamming” is observed.

In the flow and placement of concrete, it is important that the fluid concrete have the capability to move around barriers such as steel concrete reinforcements, known as rebars. Figures 9 and 10 show the flow of spherical aggregates around stationary cylinders which represent steel rebars. In one case (Fig. 9) the diameter of the spheres is on the order of one-half that of the gap between the rebars. Here the sphere becomes “jammed” as a bridge of spheres forms across the rebars obstructing the flow. For comparison (Fig. 10), when the sphere diameter is about one fifth the gap spacing (and the same volume fraction of spheres is maintained), the suspension continuously flows. No evidence of the jamming is found over the course of the simulation.

**Fig. 11:**
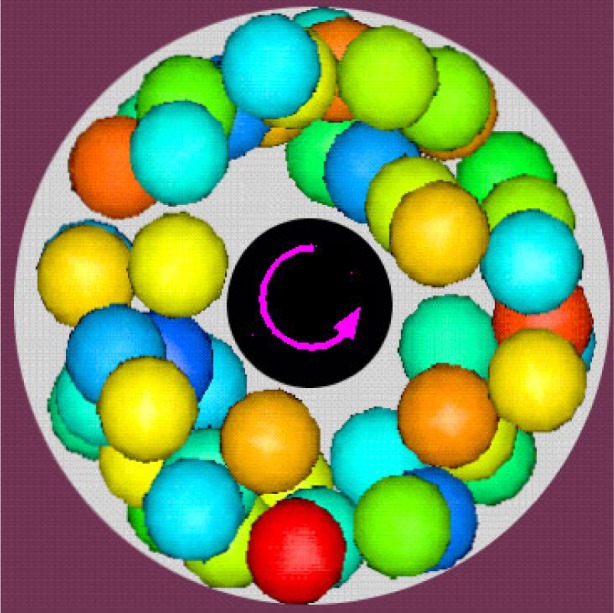
Coaxial Rheometer. An inner cylinder (in black) subject to an applied torque rotates, causing the motion of the spheres.

Figure 11 shows the motion of a suspension of spheres in a coaxial geometry. The motion of spheres is driven by the rotation of the inner cylinder. The viscosity of a suspension is often determined in a coaxial rheometer where an inner cylinder rotates as it is subject to an applied torque. Normally, knowing the torque and the subsequent rotation rate of the inner cylinder, one can derive the viscosity of the fluid in this flow geometry. However, we found that the numerical determination of viscosity can be influenced by the flow geometry in subtle ways. As can be seen in Fig. 11, the spheres had a tendency to move away from the inner cylinder. As a result the coupling between the inner cylinder and the sphere suspension was weaker so that the measurement of the viscosity was lower than anticipated. Such information can help an experimentalist better interpret measurements made with rheometers using a similar geometry and can lead to improvements in their design.

The combination of parallel processing and high end visualization (including virtual environments) has allowed us to systematically explore regions of parameter space (e.g., different solid fractions, broader particle size and shape distributions) that would be prohibitive on single processor computers.

## 5. Rapid Computation of X-Ray Absorption Using Parallel Computation; FeffMPI

Multiple scattering (MS) theory is widely used to calculate physical properties of solids, ranging from electronic structure to optical and x-ray response. X-ray absorption spectroscopy (XAS) uses energy-dependent modulations of photoelectron scattering to probe excited states and thus is important for determining electronic and chemical information from x-ray spectra [40]. XAS is usually divided into the extended x-ray absorption fine structure (EXAFS) with photoelectron energies above ≈ 70 eV, and the x-ray absorption near edge structure (XANES) in the 0 eV to 70 eV range. Theoretical calculations of photoelectron scattering are now an integral part of both EXAFS and XANES analysis. These theoretical calculations have grown in sophistication and complexity over the past twenty years. Fortunately computing power has increased dramatically (in accordance with Moore’s law [41]) during the same time period, and as a result EXAFS calculations are now fast, accurate and easily executed on inexpensive desktop computers [42, 43]. However, XANES calculations are even today time consuming for many materials. The photoelectron mean free path is large at the low photoelectron energies of the XANES region, so accurate XANES calculations require large atomic clusters and remain challenging on even the fastest single processor machines. Furthermore, the photoelectron scattering is strong for low energies, so that full multiple scattering calculations are required. These calculations require repeated inversions of large matrices which scale as the cube of the size of the atomic cluster [44]. Fortunately, parallel processing using MPI, combined with modern Lanczos type MS algorithms [45], can speed real-space XANES and electronic structure calculations by about two orders of magnitude. In particular, FEFF [46], one of the most commonly used programs for XAS analysis (developed at the University of Washington) has been improved in this manner, leading to a parallel version, FeffMPI [47].

To evaluate how well the parallel algorithm succeeds, we conducted tests on a number of systems. The results, on various systems, are tabulated in Table 2 for an 87 atom GaN test case (*N* is the number of processors, times are min).

With the improved efficiency of FeffMPI now in hand, it is feasible to carry out XANES calculations which otherwise would have been impractical. For example, a few days of calculations on a 48 processor Linux cluster can now complete a calculation that would take a year on a current single processor. Systems such as complex minerals, oxide compounds, biological structures and other nano-scale systems are obvious targets for this type of improved capability. The improved speed should be very useful, for example, for magnetic materials, which often have a large number of inequivalent sites of absorbing atoms, requiring many separate calculations to produce a full XANES or XMCD (x-ray magnetic circular dichroism) spectrum. Finally, the availability of rapid calculations now permits closed loop fitting of XANES spectra both to physical and chemical phenomena.

One interesting set of numbers are the Apple results. FeffMPI runs on PCs running Windows and Linux as well as most commercial UNIX vendor machines. The list of machines supported has recently been extended to include the Apple Macintosh running the new OS X operating system. Apple’s flagship G4 PowerMacs are powerful number-crunchers, and since its new operating system is based on UNIX, it has been attracting a lot of interest in the scientific community. Naturally there is interest in building clusters of these machines, and the needed technology has just become available with the development of Mac OS X Rack systems. For software, one can use either MacMPI [48], or a portable MPI library such as LAM [49] or MPICH [50]. The eight node G4 533 MHz results above were obtained using FeffMPI on an Appleseed cluster.

### 5.1 Results on Parallel Processing Clusters

As one example of these calculations, we show how XANES calculations can be used in the study of amorphous germanium (aGe). It is well known that the structure of amorphous tetrahedral semiconductors can be modeled well by an approach called a continuous random network (CRN). In this method, the amorphous semiconductor retains the parent structure of the crystal, but various degrees of freedom, the interatomic distance, the bond angle and the dihedral angle, are allowed to become statistically disordered [51]. Originally built by hand with ball-and-stick models, CRNs have been generated by computer, and the degree of disorder in the structural parameters is determined by energy minimization methods.

Comparisons of CRN models with EXAFS data have been done, but these comparisons were not extended into the XANES region because of the inability to perform ab initio XANES calculations, and even in the EXAFS region the calculations were limited to a simple single scattering theory [52]. Here we show that we can use FeffMPI and a CRN model to reproduce the main features in the XANES of crystalline and amorphous germanium.

XANES calculations of amorphous materials are a good example of the use of FeffMPI because, in principle, each atomic site in the CRN is inequivalent to every other site because of the statistical disorder in the model. Therefore, accurate calculations of XANES must explicitly include an ensemble average over a number of sites in the CRN in order to yield meaningful results.

As a starting point for the XANES calculation of aGe, we first modeled the XANES of crystalline germanium to determine the cluster size needed to accurately reproduce the XANES. We found that a cluster of 87 atoms, which includes the first 7 coordination shells, out to a distance of approximately 0.78 nm is sufficient to reproduce the main features of the experimental data. The primary feature that evolves as the cluster size increases is the shoulder on the high energy side of the “white line”, i.e., the first large peak in the spectra.

The aGe XANES calculations were then carried out using similar clusters of 87 atoms that had nearly the same size as the crystalline cluster, because the CRN yields a structure that has the same density as crystalline Ge, to within a few percent. Amorphous Ge is chemically and structurally very similar to crystalline Ge, so we made no adjustments in the potential determination between the two structures. The CRN model is constructed so that the atomic coordinates are expressed in multiples of the first shell bond length. In converting this to absolute distances, we assumed that the first shell Ge-Ge distance in aGe is the same as in crystalline Ge; to very high accuracy this assumption has been shown to be correct [53].

In order to get a good ensemble average over the inequivalent sites of the CRN, we ran the same Feff calculation over a total of 20 sites in the CRN.

We tested the calculation on a single processor desktop machine (with a 450 MHz CPU), where a single run took approximately one hour. We then used a 16 processor cluster (each processor having a 1 GHz CPU) where a single run took about 3 minutes. Using FeffMPI and this fairly modest cluster size thus reduced the total calculation time from 20 hours to 1 hour. We have previously studied [54] the scaling of Feff with the number of processors and found that the runtime scales as 0.03 + (0.97/*N*_p_), where *N*_p_ is the number of processors in the cluster, and a single processor runtime is normalized to 1.0. Thus, we achieve a factor ≈ 11 from the use of 16 1GHhz processors, compared to a single 1 GHz processor of the same cluster. The additional factor of two (relative to the original desktop machine) comes from the increased clockrate of the processors in the cluster.

**Fig. 12:**
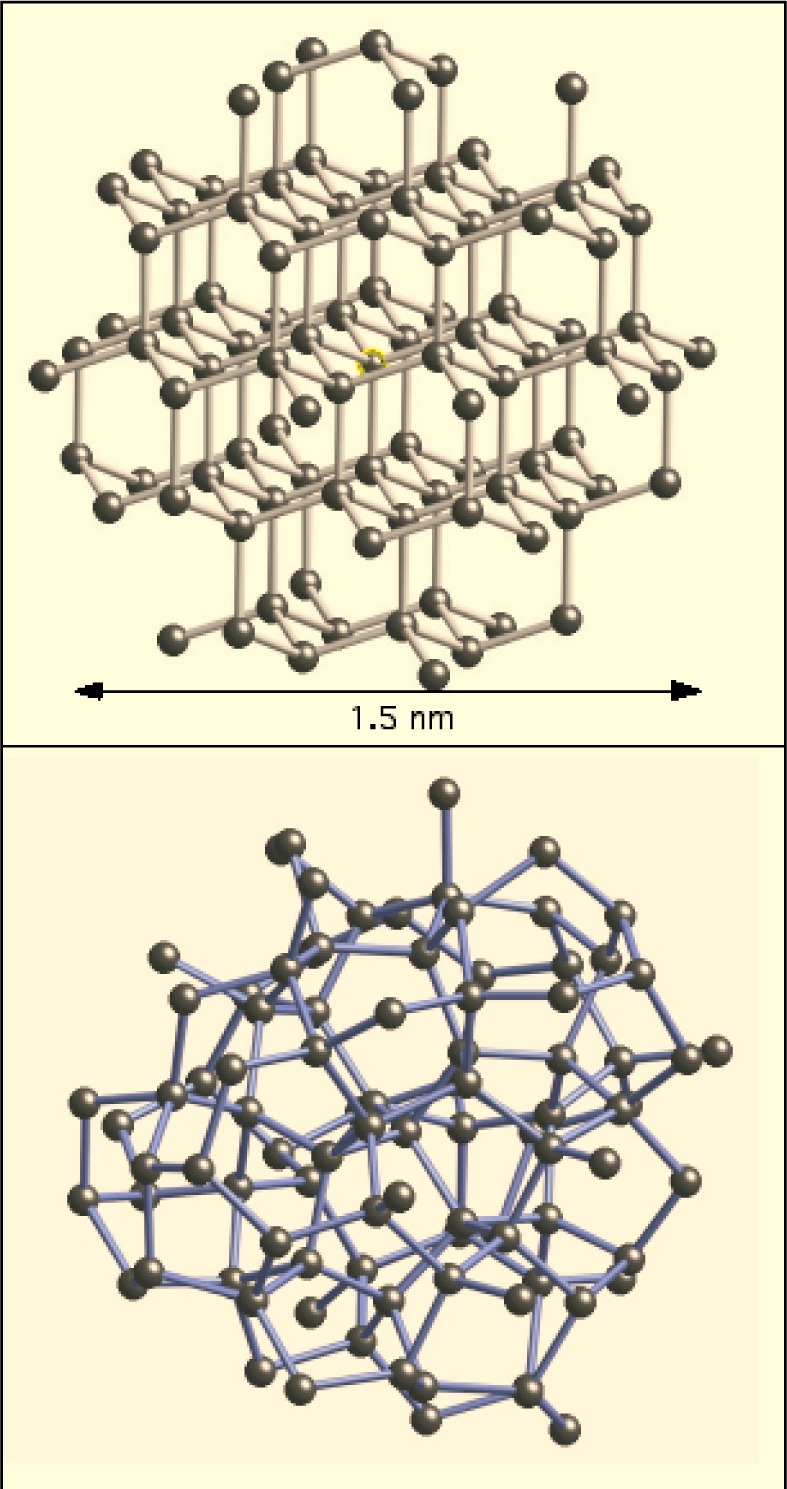
(a) The 87 atom cluster used to calculate the XANES of crystalline Ge. (b) A similar cluster of 87 atoms of aGe from the CRN displayed with the same length scale.

**Fig. 13:**
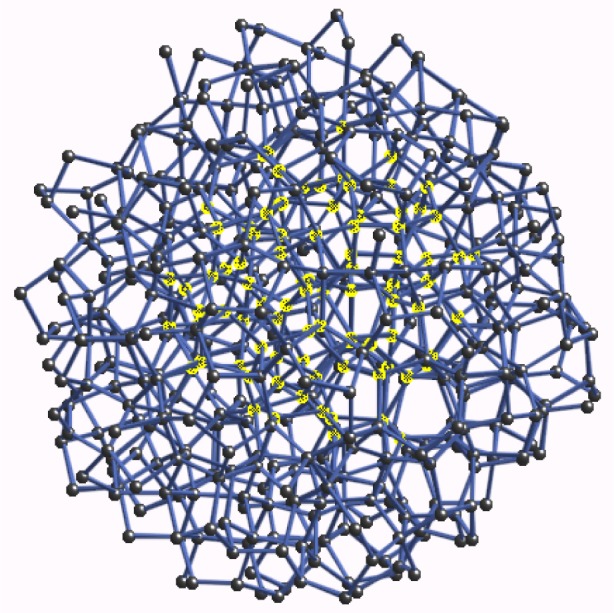
The full 519 atom cluster of aGe from the continuous random network with a typical cluster of 87 atoms highlighted in the interior.

In Fig. 12(a) we show the 87 atom cluster used to calculate the XANES of crystalline Ge. In Fig. 12(b) we show a similar cluster of 87 atoms of aGe from the CRN displayed with the same length scale. As shown in the figure, each cluster is about 1.5 nm across. In Fig. 13 we show the full 519 atom cluster of aGe from the CRN with a typical cluster of 87 atoms highlighted in the interior. Although there are several hundred atoms in the interior of the 519 atom cluster that are fully coordinated by 87 atoms, we obtain an accurate ensemble average using just 20 to 30 atoms near the center of the cluster. The convergence occurs quickly since averaging over *N* sites includes 4*N* first neighbor atoms, 12*N* second neighbor atoms, etc. The disorder in the CRN is large enough that the separation of the neighboring atoms into separate coordination shells breaks down by the third or fourth shell.

**Fig. 14:**
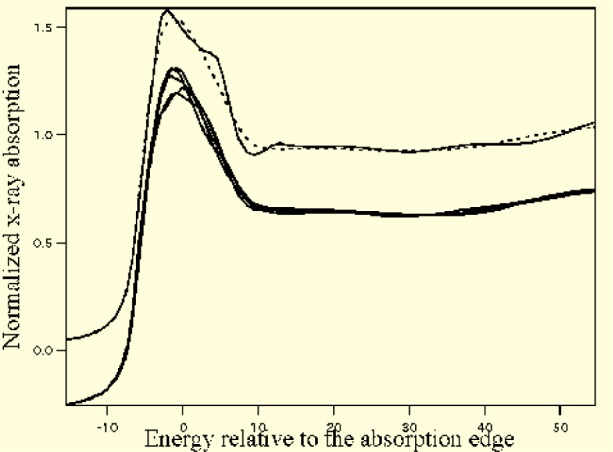
Results of the crystalline Ge calculation (upper solid line), the ensemble average over 20 sites in the aGe CRN (dashed line), and an illustration of the site-to-site variation in the aGe (five offset solid lines).

In Fig. 14 we show the results of the crystalline Ge calculation (upper solid line), the ensemble average over 20 sites in the aGe CRN (dashed line), and an illustration of the site-to-site variation in the aGe (five offset solid lines). The five single sites that are shown from the aGe calculations illustrate that there is considerable variance in the structure at any given site of the CRN, but that the ensemble-averaged XANES removes much of the structure from the spectrum, in agreement with what is observed experimentally [55].

## 6. Replicated Data Parallelizations of the Dynamics of Fluids

Understanding the atomic and molecular basis of a fluid’s properties has been greatly aided by the use of computers and is commonly referred to as molecular dynamics (MD). Often the fluid system is modeled as an assembly of rigid molecules, atoms, or ions; the forces of interaction are derived from continuous potential functions acting between (usually atomic) sites on each molecule; and the dynamics are governed by the classical Newtonian equations of motion.

We have written two different sequential programs both fitting the above model. In both cases, even with very fast modern computers, there eventually arose a need for greater speed and memory, i.e., the computer simulations were taking on the order of days for small simulations.

It has been predicted since the early 1980s [56] that parallel computers would offer the very highest performance for scientific computing. That vision has been somewhat slow in coming. Nevertheless, molecular dynamics was one of the earliest applications of parallel computers.

The emergence and widespread adoption of the single program, multiple data (SPMD) programming model and the standardization of parallel communications libraries in the 1990s have increased the use of parallel computers and offered the carrot of the very highest performance for scientific computing. Another significant advance has been the availability of the message passing interface standard MPI [37, 38]. A program can now be both parallel and sufficiently independent of architectural details to be portable to a wide range of parallel environments, including shared-memory and distributed-memory multiprocessors, networks of workstations, and distributed cluster computers. We used MPI to parallelize both of the programs discussed below.

One of the programs is a quaternion-based dissipative particle dynamics (QDPD) program which is being used to study the flow of suspensions and is discussed more thoroughly in Sec. 4.

Another of the programs attempts to model a simple two-component Lennard-Jones (L-J) fluid, i.e., defined by a Lennard-Jones potential which will be discussed later. Among the outputs of the simulation are particle density profile, potential energy profile, and stress tensor components. Since the system has two components, a surface is formed when the system is allowed to run for some time. The stress tensor components at equilibrium can be used to calculate the surface tension over this interface.

Both of the programs were originally written in Fortran 77 as serial programs. To accelerate scientific discovery, a parallelization of the program was done relatively quickly, in MPI [37, 38], using a simplified version of the replicated data approach [57]. The advantage of the replicated data approach is, first and foremost, ease of programming. Most of the parallel program is identical to the serial program which greatly simplifies program maintenance. Also, load balancing is much more straightforward than with more sophisticated methods. Last, but not least, communication consists of one global summation per timestep. Our use of the replicated data approach is discussed in greater detail below.

The starting point for “parallelizing” a serial program is to first determine which parts of the program are the most time consuming (sometimes referred to as hot spots). Before parallelizing the program, tune the hot spots. Then get the profile of the tuned serial program. On UNIX machines, standard utilities can be used to identify the hot spots. For this purpose we used Speed-Shop, an SGI integrated package of performance tools that let you run performance experiments on programs and examine the results of those experiments.

As expected on physical grounds, our profiling tests showed that the loop to compute forces dominates the time; over 90 % of the CPU time is spent inside the loop for both programs.

The basic technique of parallelizing DO loops is to distribute iterations among processes and to let each process do its portion in parallel. Both programs have a forces calculation which uses a link-cell algorithm. The MD (or QDPD) cell is divided into a number of “subcells” and the linked list structure lists the molecules or atoms belonging to each subcell. The outermost loop of the link-cell forces calculation runs over all subcells within the simulation cell. In the serial case, it has the form

do icell = 1, ncell
 … evaluate forces on particles
   in the cell
enddo
where ncell is the number of cells. In the parallel case, the iterations of this loop are distributed over processors by rewriting it as

do icell = this_process+1,ncell,nprocs
  … evaluate forces on particles
   in the cell
enddo
where nprocs is the number of processors. The only real parallel operation needed is a global sum operation to add the separate contributions to the forces and propagate a copy of the complete force array to every processor. That’s all that is needed!

We have just described the *replicated data* approach [58, 57, 59], which gets its name from the fact that every processor has a complete copy of all the arrays containing dynamical variables for every particle. The computation of forces is distributed over processors on the basis of either cell indices (as above) or particles. This is a very efficient way of implementing parallelism since the forces must be summed over processors only once per timestep, thus minimizing interprocessor communication costs. On shared-memory machines like an SGI Origin 2000, this approach is very attractive, since all processors can share the arrays containing dynamical variables.

The biggest disadvantage of the replicated data strategy is that every processor must maintain a copy of all of the data, and therefore the data must be updated on each processor at the end of each time step. This is not a problem in the shared-memory multiprocessor version if the MPI implementation is smart enough to take advantage of the shared memory (which is the case on the SGI Origins). Using this technique, there is no need to distribute particle data since each processor has a copy of all of the data and updates them all; this approach has worked well for small to medium sized problems (tens-of-thousands of particles) on the shared-memory SGIs. An example is our Lennard-Jones fluid program which uses MD simulation to gain insight into the choice of various order parameters and correlation functions which are associated with fluid-fluid interfaces, liquid films on solid substrates, and surface-tethered monolayers of chain molecules.

Dynamics of interfaces are challenging because the wide variety of rate phenomena that are encountered at interfaces requires a high level of sophistication in dealing with them. But the payoff in understanding these phase changes and fluctuations is a better understanding of rates of evaporation, condensation, absorption and desorption, dissolution, and precipitation, among other things.

**Fig. 15:**
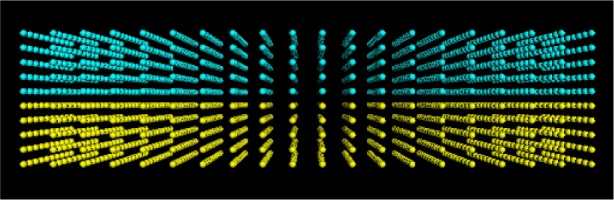
Snapshot of initial configuration of a two-component fluidfluid interface system. One component is shown in blue and the other component is shown in yellow.

Our two-component fluid-fluid interface system consists of 2000 (1000 per component) particles interacting by a Lennard-Jones potential

$$V_{\text{LJ}}=4\cdot \varepsilon [{\left(\sigma /r\right)}^{12}-{\left(\sigma /r\right)}^{6})]$$ (2)

where *ϵ* and *σ* are the energy depth and range parameters, respectively. In addition to the Lennard-Jones potential, there are three interactions which contribute to surface or interfacial formation, two for the two different like-particle attractions and one for unlike particle repulsion. The system, which could be, for example, oil and water (or any two relatively immiscible fluids), has periodic boundary conditions and is initially configured as a lattice as shown in Fig. 15. The two components are evenly distributed throughout their separate volumes prior to mixing.

**Fig. 16:**
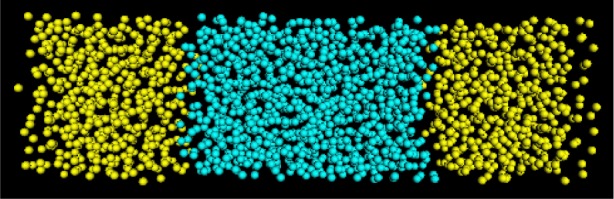
Two-component fluid-fluid interface system at equilibrium. As seen in the figure, equilibrium is characterized by two distinct interfaces.

Though the figure shows particles of distinct size, in the program the particles are simulated as points. This is equivalent to particle interaction distances being measured from particle centers for particles of distinct size. Once the system is “turned on”, it is allowed to run until thermal equilibrium is reached (Fig. 16). In our calculations, we consider the system at equilibrium if the kinetic energy fluctuation is small. At that point (equilibrium), we collect data on the system. Since the interaction is on the femtosecond (10^−15^ s) scale, we consider a run of 10 picoseconds (10^−12^ s) to be a long one.

**Fig. 17:**
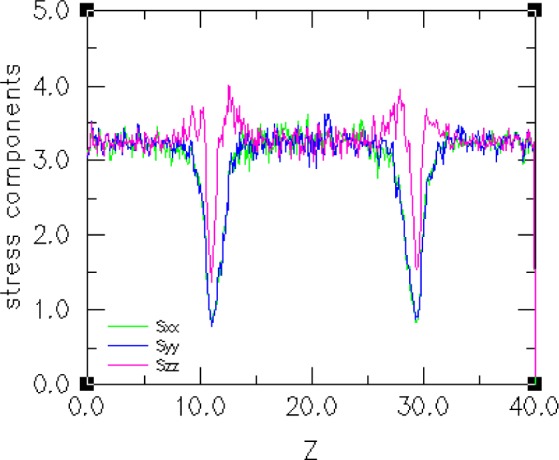
Plot of the *x*, *y*, and *z* components of the stress tensor. As can be seen from the distinct dips in the curves, the greatest change in these components occurs in the interfacial regions.

**Fig. 18:**
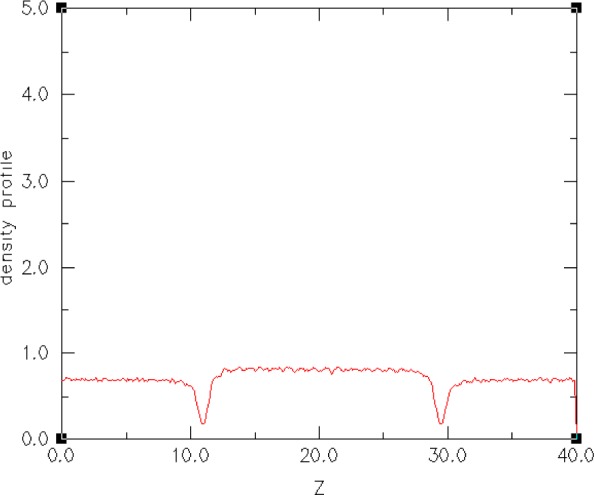
Plot of the particle density. The fact that the unlike particles do not like to mix is evident from the distinct decrease (the dips in the curve) in the particle density in the interfacial regions.

In addition to determining the microscopic surface density profiles of various atomic species, we would like to be able to extract the components of the molecular-level Kirkwood stress tensor [60] from the simulation of a small selected set of hydrophobic-hydrophilic interfaces and make comparisons of their relative surface tensions. Our reason for doing this is that Kirkwood stress tensor integration is a way to determine surface tension (another technique is density functional theory [61]). In Fig. 17 the stress tensor profile is shown at equilibrium. Fig. 18 shows a density profile at equilibrium. A density profile in the initial configuration (Fig. 15) of two separate components would be flat, since the two separate components are completely mixed (and at equilibrium) prior to bringing them together and allowing them to attain the new equilibrium of the two-component system. In contrast, in Fig. 18 the fact that unlike particles do not like to mix is evident from the distinct decrease (the dips in the curve) in the particle density in the interfacial regions, and provides a quantitative measure of what the visualization in Fig. 16 shows.

Kirkwood stress tensors and density functional theory are two ways to determine surface tension. Our plans are to investigate the use of a third method, a thermodynamic titration method (a process to connect heat flow for interfacial production or destruction to surface tension) to derive the surface tension. This should result in a flexible, convenient, and reliable method to check experimental as well as theoretical approaches to surface tension studies. It should also help expand the current data of this area of surface science.

By parallelizing the program, the L-J fluid simulation run time decreases by a factor greater than six. This allows more flexibility in starting, checking, and analyzing runs. It also provides a means for more accurately determining the behavior of surface tension, pressure, and potential profiles.

In the case of QDPD, the parallelization using the above replicated data has been the workhorse for some time now. However, it has had its disadvantages. The biggest disadvantage of the replicated data approach is that it tends to get communication bound as the number of processors increases. Scaling to very large numbers of processors in a shared-memory environment is poor (24 is the practical limit for us), and it has turned out to be almost unusable on distributed memory systems including those with high speed interconnects like the IBM SP2/SP3 systems.

The solution to these problems is to do a spatial decomposition as we will describe fully in a future publication [39].

## 7. Nanotechnology and Parallel Computing

**Fig. 19:**
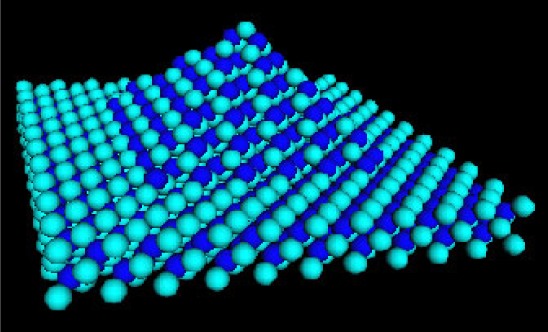
Pyramid structure.

Accurate atomic-scale quantum theory of nanostructures and nanosystems fabricated from nanostructures enables precision metrology of these nanosystems and provides the predictive, precision modeling tools needed for engineering these systems for applications including advanced semiconductor lasers and detectors, single photon sources and detectors, biosensors, and nanoarchitectures for quantum coherent technologies such as quantum computing. The tight-binding model [62] based upon the Linear Combination of Atomic Orbitals (LCAO) method provides an atomistic theory for nanostructures which is accurate and easy to implement [63]. The tight-binding method is ideal for modeling small nanostructures [64], for example the pyramid shown in Fig. 19. However, for modeling nanostructures with more than 25 000 atoms, the method is impractical on sequential computers due to long run times. Significant improvements in run time can be achieved through parallelization.

### 7.1 Parallelization

There are two parts to parallelizing this problem: creating the structure; and solving the Hamiltonian equation.

**Fig. 20:**
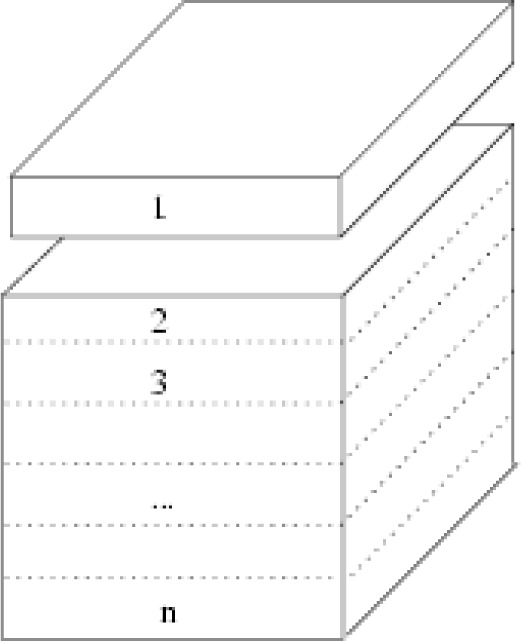
A distribution of the computation via layers.

The structure is created geometrically. We parallelize this [65] by dividing the structure into layers. See Fig. 20. Communication is across layers. The starting point is a cubic structure that encompasses the desired nanostructure; the structure shape is created by pruning away the excess.

Once the atom positions are determined, then the Hamiltonian is determined. Once the Hamiltonian is known, it is diagonalized to find the electron eigen energies and eigen states, i.e. the electron energies and charge distribution at each atom site. This is parallelized with PARPACK [66].

**Fig. 21:**
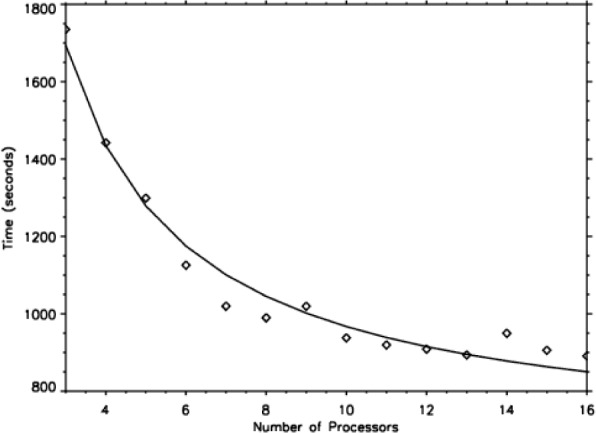
Execution time versus number of processors for concentric spheres problem.

We ran the code on the NIST NBS Cluster of 500Mhz Pentium III processors. Each processor has a Gibibyte, i.e., 2^30^ bytes of memory. Figure 21 shows performance data for three concentric spheres with diameters 3, 4, and 5 lattice units. This data matches closely the formula: *T* = 655.7 + 3116.0/*N*. *T* is execution time (in s), and *N* is the number of processors. The non-parallelizable computation time is 655.7 s; while the parallelizable portion of the computation uses 3116.0 s. Thus the portion of the code that was directly parallelizable with PARPACK is almost 83 %.

### 7.2 Visualization

**Fig. 22:**
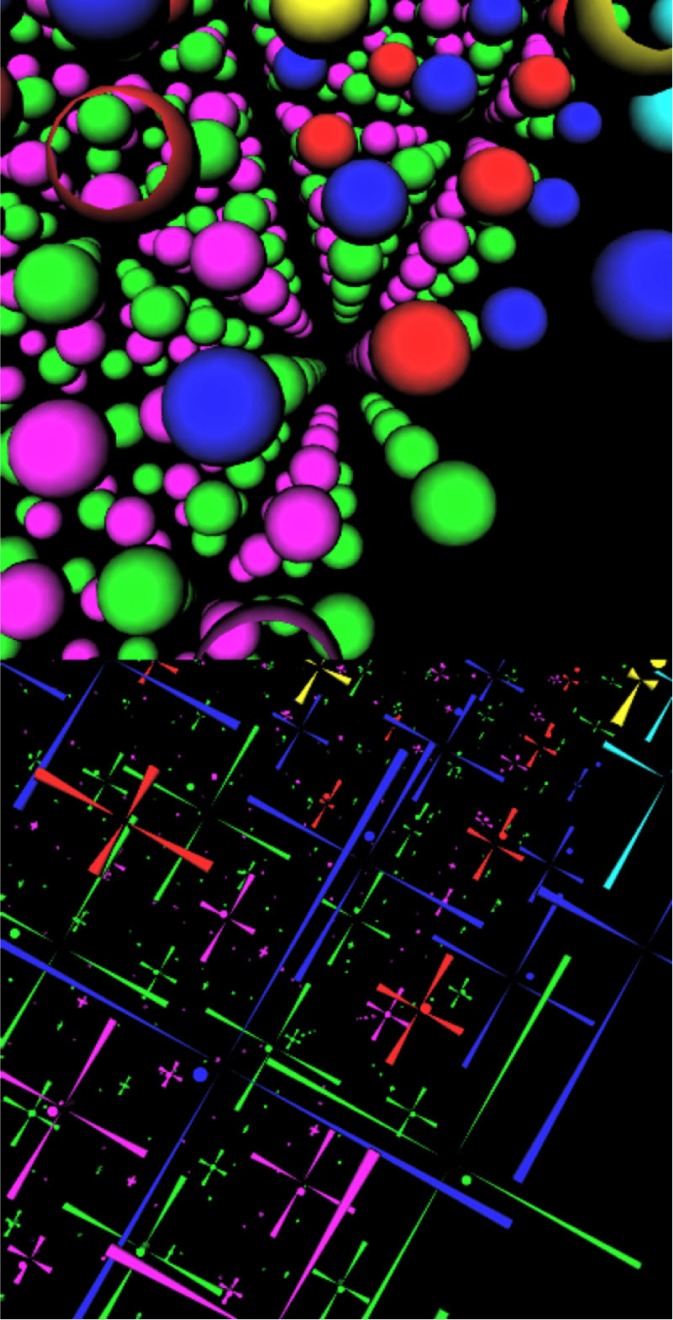
A section of a nanostructure (top) with orbitals (bottom).

See Figure 22 for a visualization of a cross section of a nanostructure (top) with orbitals (bottom). The program output is transferred to the NIST immersive environment where the structure is studied interactively. This provides a detailed visualization of the structures and the atomic scale variation of calculated nanostructure properties that is not possible with any static graphical representation. We save the interaction with the structure in the immersive environment as a quicktime movie. The parallel implementation can handle arbitrary nanostructure shapes through an input file specification procedure.

## 8. Dendritic Growth in Alloys

Our simulation of the solidification process for a binary alloy is implemented as a parallel C program, using MPI for interprocessor communication. The output from this simulator consists of a series of snapshots of a volume of material, consisting of two metals, taken as it solidifies. This material is initially in a supersaturated liquid state except for a small seed of solid required to initiate the solidification process. Detailed descriptions of this simulator have been previously published [67, 68].

Thanks to recent updates to our available computing resources we are now able to extend our simulations to uniform finite-difference grids of size 1000 × 1000 × 1000 using 70 or more compute nodes from our 128-node Beowulf cluster. This has been a goal of this project since smaller simulations do not reveal sufficient detail or produce dendrites sufficiently developed to fully exhibit relevant properties. Also, prior 2D dendritic growth simulations on grids of size 1000 × 1000 can be directly compared to these results.

At this point, the limiting factor in this project is the visualization of the resulting 3D snapshots. Currently available commercial and open source visualization software has so far been incapable of processing these snapshots, mostly due to internal array indexing limitations and memory addressing issues, especially while computing isosurfaces. Also, interactive display of full resolution rendered images of the dendrites from simulations on grids of 300^3^ or larger has been too slow for practical use. As part of our research effort, we are exploring techniques to enhance our capabilities in these areas, for this particular application as well as other large simulations.

**Fig. 23:**
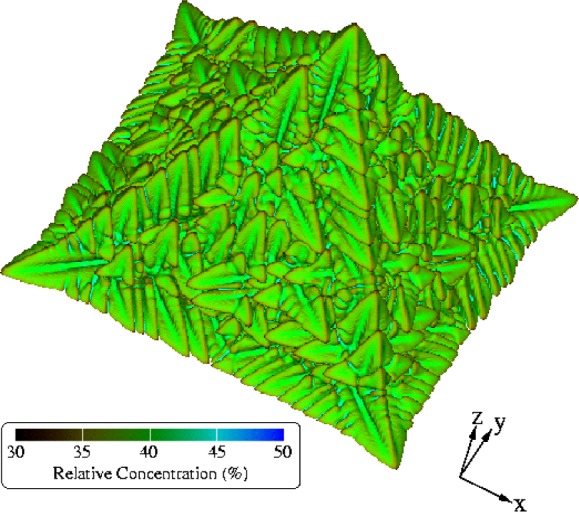
A simulated copper-nickel dendrite computed over a uniform 3D grid of 500^3^ points. Two of the axes have been mirrored resulting in this image of 1000 × 1000 × 500 (*x*, *y*, *z*) points. The physical dimensions of this dendrite are approximately 35 μm by 35 μm by 17.5 μm. The color of the dendrite indicates the concentration of copper in the copper-nickel alloy at each point on the surface.

To date, the largest dendrite that we have rendered has been from a simulation over a uniform finite-difference grid of 500^3^ points. Figure 23 shows one image of this dendrite taken near the end of the simulation. Typically the raw data from the snapshots are reflected across all three physical dimensions to produce a six-pointed object. However, because of limitations of the visualization software, this image has been reflected across only the *x* and *y* axes, yielding the five-pointed object shown. The surface of the dendrite is colored according to the relative concentration of the two metals that comprise the alloy, copper and nickel. This aspect of the alloy, the relative concentration of the constituent metals, is an important factor in determining the final properties of the alloy. The key in Fig. 23 indicates the mapping of surface color to the percentage of copper in the material.

**Fig. 24:**
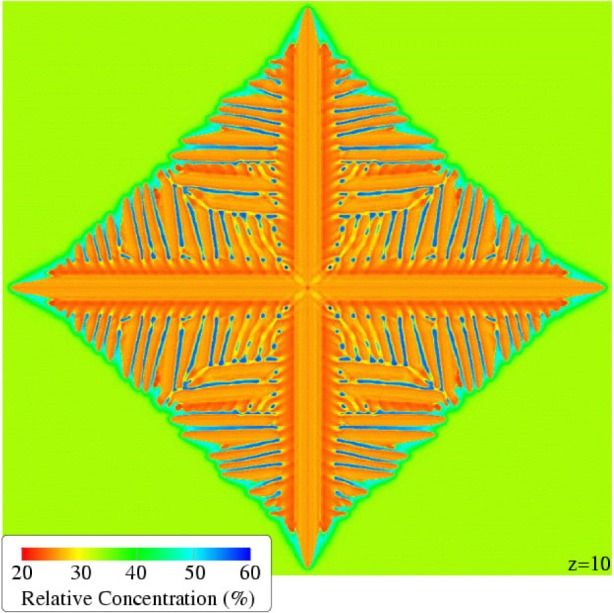
A 2D (*x*, *y*) slice near the base of the dendrite shown in Fig. 23.

**Fig. 25:**
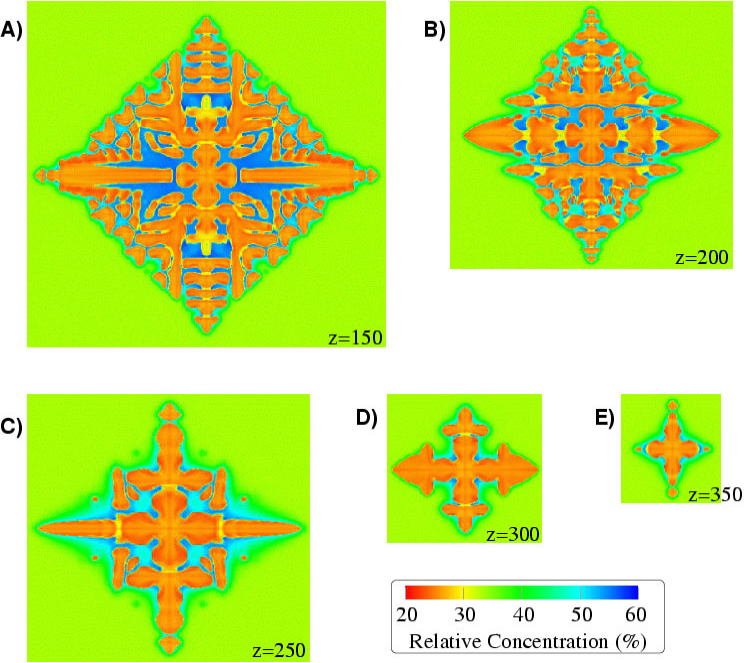
Several more 2D slices of the dendrite in Fig. 23. Each *z* value indicates by an integer the position of the slice along the *z* axis.

By taking 2D slices through the dendrite, the internal structure of the dendrite can be inspected. Figures 24 and 25 show some of the detail of the dendrite, specifically the distribution of the constituent materials, with 2D slices taken at several points along the *z* axis. Although the images in Fig. 25 are of different sizes, they are all shown at the same scale as Fig. 24, only the surrounding area, which is still in the liquid phase, has been clipped to save space.

Further efforts should enable us to render the output from these simulations on 1000^3^ grids and to interactively display these images on systems ranging from high-performance graphical workstations to 3D immersive environments.

## 9. Screen Saver Science

The *Screen Saver Science* (SSS) project is a research project to develop a parallel computing environment using any existing personal computers and scientific workstations connected to the local network as the compute nodes. Each compute node would perform computations, as part of this computing resource, only when it would normally be idle or running a screen saver program. Optionally, the screen saver could still be running in addition to the computation.

In contrast to well known distributed computing projects, such as SETI@Home [69] (Search for Extraterrestrial Intelligence), Folding@Home [70, 71] (protein folding), Distributed.net [72] (finding prime factors, cipher cracking), and others, the clients of this system, that is, the part that runs on the user’s workstation, will not consist of a dedicated scientific application. In the SSS system, the client will have no particular calculation embedded in it at all, but instead will be capable of performing any computation, subject to local resource constraints such as the amount of memory available. This is made possible through the use of applications compiled to portable Java bytecode along with the Jini and JavaSpaces technologies [73] that have been enabled by the Java environment. Another fundamental difference between SSS and other distributed computing projects is that SSS clients can communicate with each other during the computation in order to coordinate the computation, rather that simply exchanging data with a central job manager, thus presenting a distributed parallel computing model to the SSS application programmer.

This parallel coordination between the compute nodes is feasible only because the pool of participating compute nodes will all reside within the local (NIST) network, which spans the two main campuses at Gaithersburg, Maryland and Boulder, Colorado. This also greatly simplifies the design of SSS with respect to security since interprocessor communication will not need to cross any network firewalls. Compared to other distributed computing projects which can potentially utilize millions of personal computers connected to the Internet, this will limit the number of available compute nodes to a more modest value, possibly up to 3000 nodes.

The basic design for this form of a generic distributed compute server has been described by Freeman, Hupfer, and Arnold in the text *JavaSpaces Principles, Patterns, and Practice* [73]. The SSS project seeks to expand on this basic idea to produce a robust production quality distributed computing environment. This environment will also be used as a testbed for the study of parallel distributed algorithms for scientific applications.

**Fig. 26:**
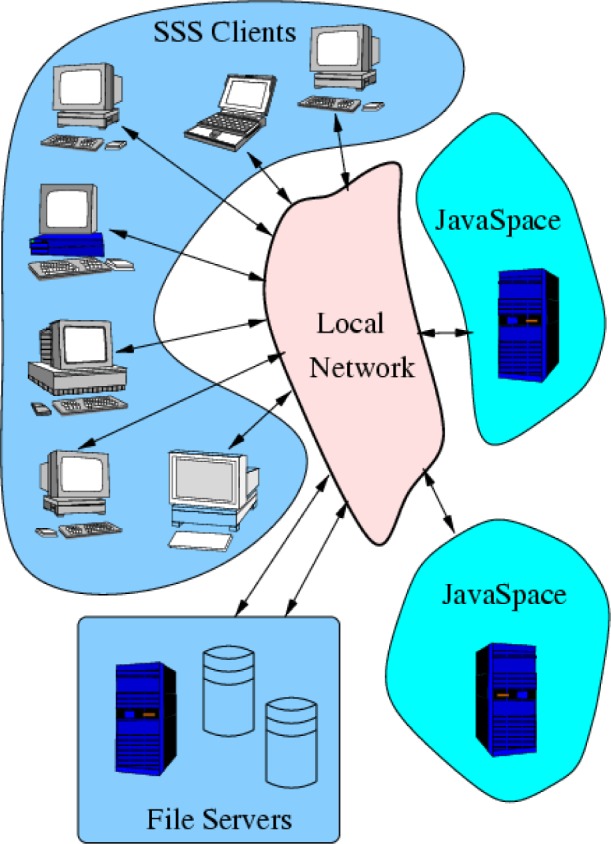
A high-level view of the Screen Saver Science architecture. Clients access a shared object space (JavaSpace) to retrieve tasks to perform, return results, send messages to other clients, broadcast status messages for general use, and exchange other messages as needed. Large data sets will be stored on disk with client access to these files coordinated through entries in the shared object spaces.

The high-level design of SSS is shown in Fig. 26. SSS clients access the shared objects (data and programs) held in the JavaSpace services and also have access to file servers for input and output of large data sets. The basic idea is that tasks (requested computations) are placed in a JavaSpace. Clients take these tasks, one at a time, and complete them. As each task is completed, the client writes the results back to the JavaSpace. Because these shared object spaces are not suitable for holding large amounts of data, one or more file servers are also available for use during these computations.

This project is in its early stages with initial design and implementation scheduled for the summer of 2002. Many issues will need to be considered in designing the application programming interface (API) for the applications programmer to use when, for example, the program needs to read or write a file, or to return a result to the shared object space. Many other issues not directly related to the implementation of SSS applications need to be addressed also such as the design of the *space objects* that will carry information to and from the clients, the interface between the SSS client and the native screen saver, and supplementary applications for monitoring the status of the environment.

The initial application targeted for this environment is a quantum Monte Carlo (QMC) computation, loosely based on a Fortran 77 QMC application (Quantum MagiC) [74, 75]. This computation was chosen because it can be implemented with a highly parallel algorithm that closely matches the model presented by the SSS computing environment. Additionally, the size of the individual tasks in a parallel QMC computation can easily be tuned to the memory size and processor speed of the available compute nodes. Also, the large number of floating-point operations required to complete a typical QMC computation makes this an ideal application for SSS.

## 10. The NIST Genetic Programming Project

Genetic Programming (GP) is a technique for the automatic design and implementation of algorithms. We are building a generic, parallel, genetic programming system that can be used on a variety of real-world scientific problems.

The user specifies the problem to be solved and provides the building blocks; the system determines an algorithm that fits the building blocks together into a solution to the specified problem.

### 10.1 Background

Genetic programming is a technique that is inspired by the processes of biological evolution. A population of programs evolve toward a solution to a given problem under the pressure of a *survival of the fittest* mechanism. Genetic programming may be thought of as a type of machine learning that automatically can derive an algorithm from a relatively simple problem specification.

More specifically, an initial population of randomly constructed programs is created and each individual program is evaluated to determine how well it solves the given problem. In all likelihood, in this initial population, all of the programs will be very poor solutions to the given problem, but some will be slightly better than others. A new population of programs is then derived from the old population. This is done by creating new individual programs from the old programs by biologically-inspired transformations referred to as mutation, crossover, and survival. Preference in these operations is given to the individual programs that are judged to be better solutions to the given problem. It is this preference for the *fittest* programs that causes the population to evolve toward better solutions.

The new population is called the next generation. This process of evaluation of the fitness of the individual programs and the creation of the next generation based on the best programs from the old generation is then repeated until a satisfactory solution is found.

It should be noted that the process of measuring how well a program solves a given problem is of critical importance. This fitness measurement procedure embodies the problem to be solved. It determines the landscape that the GP search algorithm traverses and applies the force that causes the population of programs to move toward a solution.

### 10.2 The NIST GP Implementation

We have implemented our GP system [76] using a somewhat unconventional representation for individual (evolvable) programs. We have used a program representation that is modeled on the higher level programming languages typically used by human programmers, such as C or Fortran. This is in contrast to the LISP-like representation that is often used in genetic programming systems [77, 78, 79]. We describe our program representation as a *procedural* representation in order to denote the structuring of the program into a hierarchy of routines, each composed of a sequence of calls to other routines.

We expect that this representation, with program structures that are useful to human programmers, may prove to be useful in evolved programs. Furthermore, although the same programs can be expressed in either the LISP-like syntax or in our procedural structures, the evolutionary characteristics will certainly be different. Hence the procedural representation could yield better results for some problems. Of course, by the same token, the procedural representation could yield worse results in other cases [80]. This suggests that these two representations (and perhaps others) might be used together in a single system. We are exploring this idea in a related project [81].

### 10.3 Results

**Fig. 27:**
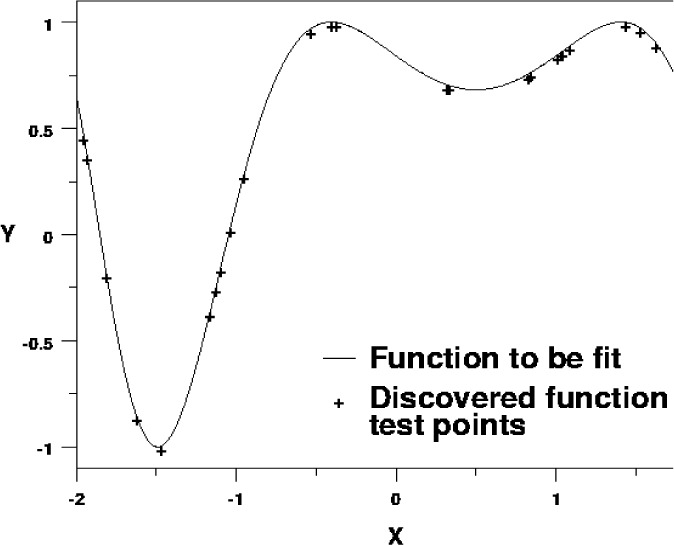
Results of an example regression run. Values of the GP-discovered function are shown at points that were used by the GP system to determine fitness.

We are studying symbolic regression with our GP system. Figure 27 shows the results of a GP run on one problem. The function that is being fitted is sin(*x*^2^ + *x* + 1). The figure depicts a portion of the function, and the values of the fitted function at some of the test points that the GP system used during its fitting. Clearly the GP system closely matched the target function. In fact, our GP system found the function cos(*x*^2^ + *x −* 0.570562). Note that the GP system found and used the constant 0.570562, which is a close approximation to π/2 − 1. It then exploits the fact that sin(*a*) = cos(*a −* π/2) to fit the data from the original function.

One goal of the regression project is to automatically find functional forms for measurement errors in combinatorial experiments.

In conjunction with our procedural program representation we have also introduced two new operations, *repair* and *pruning*, that are designed to work with the peculiar properties of our program representation. These operations are used much in the manner of mutation or crossover to generate new programs from existing programs in the evolving population of programs. Both of these operations have had substantial, and sometimes unexpected effects on the system’s effectiveness.

The *repair* operation scans selected programs and looks for cases of unused variables or results, unset output arguments, and the use of uninitialized variables. When one of these conditions is found, the program is automatically altered to eliminate the situation. We have found that these *repairs* can enable the GP system to more easily evolve a population toward a solution.

The *pruning* operation goes through a program and attempts to cut off as many program branches as possible without hurting the fitness of the program. Pruning was initially implemented to address the problem of program *bloat*. This is a common problem in GP systems; it is the situation in which program size grows unmanageably large, causing extreme slow-down of evolution. Techniques for reducing bloat have been studied [82].

Our pruning operation goes through a selected program and attempts to cut off as many program branches as possible without hurting the fitness of the program. This form of pruning seems to be unlike many previous attempts to control program bloat. We found that this operation was not only quite effective in controlling program size, but that it also improves our GP system’s ability to evolve solutions. We have observed cases in which the pruning operation has acted as a type of mutation that produces a program that is substantially more fit than the program that it started with. At times, pruning has been observed to produce the most fit individual of the generation. This is an unexpected and intriguing result and we will be investigating this effect in more detail.

### 10.4 Visualization

In order to understand the operation of our GP system we instrumented the system in a variety of ways. Statistics are accumulated for program and populations such as the number of operations and variables used, program ancestry, and the type of genetic operations used. These data enabled us to gain an understanding of the operating characteristics of the system, but the volume of data is often unwieldy.

This motivated the implementation of a visual representation of populations and individual programs. These visualizations do not capture all aspects of the programs or all of the statistics mentioned above but they do provide a way of looking at broad characteristics or patterns within populations.

**Fig. 28:**
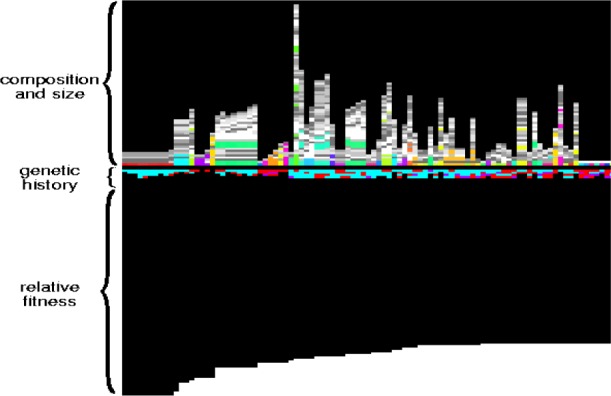
Visualization of a population.

Figure 28 shows a visualization of a population of 128 individual programs. Each program is represented by one vertical column. As indicated in the figure, three aspects of each program are represented. The upper part is a visual representation of the content of the program. Each block of color in this section corresponds to a procedure in the program. The basic operations of the system are each given a different grey level and composite procedures (which assemble sequences of the basic operations) are assigned a color that indicates ancestry. In the middle section, the sequence of genetic operations that brought each individual into existence is presented. Each operation, such as crossover and mutation, is given a unique color. Finally, the lower portion of the image presents a normalized view of the fitness of each individual. In Fig. 28, the individuals have beens sorted by fitness with the most fit individuals on the left.

**Fig. 29:**
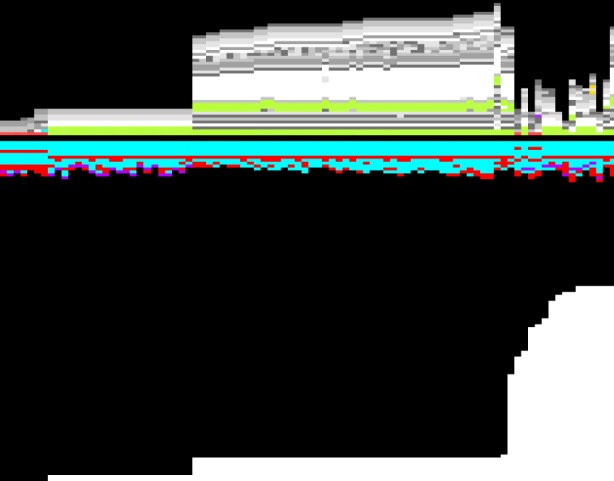
A population showing a substantial loss of diversity.

These visualizations have proven useful in several ways. For example, Fig. 29 shows the same population as that in Fig. 28 but at a later generation. One observes large numbers of programs that have very similar structures and very similar genetic histories. This visual representation clearly shows a loss of diversity in the population between the generation represented by Fig. 28 and the generation shown by Fig. 29. This has prompted us to undertake an ongoing investigation into the issue of population diversity and its influence on the operation of our GP system.

## Figures and Tables

**Table 1:** Comparison with previous explicitly correlated calculations for 1^1^S He-like ions

Author	*N*	Energy (a.u.)
Li^+^		

TK [31]	308	−7.2799 1341 2669 3020
Drake [20]	2114	−7.2799 1341 2669 3059 0000
This work	4284	−7.2799 1341 2669 3059 6489

Be^++^		

TK [31]	308	−13.6555 6623 8423 5829
Drake [20]	2114	−13.6555 6623 8423 5867
This work	4648	−13.6555 6623 8423 5867 0206

B^+3^		

TK [31]	308	−22.0309 7158 0242 7777
Drake [20]	2114	−22.0309 7158 0242 7815
This work	4648	−22.0309 7158 0242 7815 4163

**Table 2:** FeffMPI 87 atom GaN test case on various systems. Time is in minutes: seconds

System	MHz	*N*	Time
IBM SP II nighthawk		32	:27
IBM SP NERSC R6K proc		32	1:20
SGI Origin2000 R10K	250	8	2:04
Athalon	1000	4	3:09
CRAY T3E alpha EV-4 proc		8	4:12
Apple G4	533	8	4:14
Dual ALPHA EV-6	500	2	4:43
ALPHA EV-6	500	1	7:23
Dual Athlon SMP	860	2	7:51
SGI Octane R10K	250	1	14:46
Dual686 P3 Linux	450	2	15:31
Mac G4 linux	400	1	22:40
Dual686 P3 linux	450	1	27:00

## Footnotes

1 Certain commercial equipment, instruments, or materials are identified in this paper to foster understanding. Such identification does not imply recommendation or endorsement by the National Institute of Standards and Technology, nor does it imply that the materials or equipment identified are necessarily the best available for the purpose.

2 The atomic unit of energy is chosen as $\frac{\mu e^{4}}{\hbar^{2}}=1$ a.u. (of energy), where ***μ*** = *m*_e_*m_N_*/(*m*_e_ + *m_N_*).

3 See King [9] for a review, and Godefroid, Froese Fischer and Jönsson [19] for the most recent work.
